# Multicenter Study of the Seroprevalence of Antibodies against Covid-19 in Patients with Lymphoma: An Analysis of the Oncological Group for the Treatment and Study of Lymphomas (Gotel)

**DOI:** 10.3390/curroncol28020118

**Published:** 2021-03-16

**Authors:** Fernando Franco, María Guirado, Natividad Martínez-Banaclocha, Josep Gumà, Javier Lavernia, José Gómez-Codina, Delvys Rodriguez-Abreu, Fani Martínez, Enrique Barrajón, Miriam Méndez, Virginia Calvo, Mariano Provencio

**Affiliations:** 1 Medical Oncology Department, Hospital Universitario Puerta de Hierro, 28222 Majadahonda, Spain; mendez.garcia@salud.madrid.org (M.M.); virginia.calvo@salud.madrid.org (V.C.); mariano.provencio@salud.madrid.org (M.P.); 2 Medical Oncology Department, Hospital General Universitario de Elche, 03203 Elche, Spain; maria.guirado@ciemat.es (M.G.); ebm@oncologia2000.com (E.B.); 3 Medical Oncology Department, Hospital General Universitario de Alicante, 03010 Alicante, Spain; noemartinezban@gmail.com; 4 Medical Oncology Department, Hospital Universitari Sant Joan de Reus. URV. IISPV, 43204 Reus, Spain; jguma@grupsagessa.com (J.G.); fmartinezma@grupsagessa.com (F.M.); 5 Medical Oncology Department, Instituto Valenciano de Oncología, 46009 Valencia, Spain; jlavernia@fivo.org; 6 Medical Oncology Department, Hospital Universitario la Fe, 46026 Valencia, Spain; jose.gomez-codina@uv.es; 7 Medical Oncology Department, Hospital Universitario Insular de Gran Canaria, 35016 Las Palmas de Gran Canaria, Spain; delvysra@yahoo.com

**Keywords:** lymphoma, infection of SARS-CoV-2, Covid-19

## Abstract

The new Severe Acute Respiratory Syndrome Coronavirus 2 (SARS-CoV-2) coronavirus has generated a pandemic, in which there are population groups at higher risk and who are potentially fatal victims of the disease. Cancer patients have been considered a group with special susceptibility, particularly patients with lung tumour involvement and haematological neoplasms. The Spanish Lymphoma Oncology Group (GOTEL) carried out a multicenter study of SARS-CoV-2 seroprevalence in patients with lymphoma. Results: A total of 150 patients were included between 22 May and 11 June 2020. The mean age was 65 years (range 17–89), 70 women (46.5%) and 80 men (53, 5%). At the time of diagnosis of lymphoma, 13 cases were stage I (9%), 27 (18%) stage II, 37 (24.5%) stage III, and 73 (48.5%) stage IV, while 6.6% had a primary extranodal origin. A total of 10 cases with positive serology for SARS-CoV-2 were identified, which is a prevalence of 6% in this population. None of the patients required intensive care unit management and all fully recovered from the infection. Conclusion: IgG antibody seroprevalence in lymphoma patients appears similar to that of the general population and does not show greater aggressiveness.

## 1. Introduction

In late 2019, an outbreak of a new form of pneumonia was described in a group of patients in Wuhan, China [1,2]. The cause of this new entity (COVID-19) is a β-coronavirus that has been called severe acute respiratory syndrome coronavirus 2 (SARS-CoV-2) [3]. The contagion potential of the virus is such that its spread throughout the world has been rapid and has infected more than 111 million people, thus constituting the most recent pandemic declared by the World Health Organization (WHO) [4]. Although more than 80% of the cases present few or no disease symptoms [5,6,7], about 15% of the patients have a serious disease that requires hospitalization with medical support and oxygen therapy. Only 5% of patients, affected by the disease, require intensive care management given the extreme severity of the disease, determined by severe respiratory failure, septic shock, and multi-organ failure [8]. The mortality rate from covid-19 varies significantly between the different age ranges and comorbidities of the patients, ranging from 0.031% in young people to more than 7% in those over 75 years of age [9]. The high rate of spread of the disease and the speed at which it occurred created chaos in health systems around the world due to the lack of capacity of countries to combat the health crisis.

After the cases reported in China, the second main focus of the pandemic was in Europe, initially hitting severely countries, such as Italy and Spain. In Spain, more than 3.1 million people were confirmed, with more than 67,000 of them dying [10]. So far, the only measures that have proven to be effective in preventing and controlling community transmission are the active search for cases and their containment, social distancing, and the identification of populations at risk.

Cancer patients have been considered to represent a group of special susceptibility, not only due to the type of malignant disease but also due to the possible treatments used in the control of malignant neoplasms [11]. It is probable that the immunosuppression, generated by the disease or by cancer treatments, may condition a greater risk and susceptibility of patients to serious complications [12]. It is also important to note that a large number of cancer patients have advanced age and comorbidities that can contribute decisively to the evolution of COVID-19 in this population [13]. Additionally, other factors that may influence are interruptions or limitations in access to treatments derived from quarantine measures and difficulties in routine medical care due to the pandemic and to the problems in the access to diagnostic tests [14].

The analysis of population-based studies of collaborative groups and national registries of cancer patients has been able to determine patients with a higher risk of developing serious SARS-CoV-2 infections. Among them, we can highlight patients with pulmonary neoplasms or patients with high-grade haematological neoplasms (acute leukaemia and high-grade lymphomas) [11,15]. Bearing in mind that lymphomas are a wide variety of neoplasms with potentially curative treatments regardless of the stage at diagnosis, we have considered it important to carry out a study of SARS-CoV-2 seroprevalence in our population to determine the incidence in our environment.

## 2. Material and Methods

This is a multicentre and prospective study of SARS-CoV-2 seroprevalence –LINFO-COVID, carried out in medical centres attached to the Oncological Group for the Treatment and Study of Lymphomas (GOTEL) in Spain. The study protocol was evaluated and approved by the Ethics Committee at the Puerta de Hierro University Hospital in Majadahonda. The total expected sample was 150 patients diagnosed with lymphomas and followed up in the services of medical oncology, on whom a serology test was randomly performed to determine the presence of antibodies against the SARS-CoV-2 virus (IgG) through an Enzyme-Linked ImmunoSorbent Assay (ELISA) technique in peripheral blood. The variables analysed included demographic and clinical data (age, sex, histological type, stage, treatments, and current situation of the disease), showing if they had developed symptoms suggesting a SARS-CoV-2 infection and the type of treatment if they had been treated for the infection. All the patients were previously informed of the study and signed the informed consent to obtain the sample and the analysis of the clinical data for the presentation of the cases in the field of scientific discussions, respecting the data protection laws and regulations in force according to the Organic Law 15/1999 of the Protection of Personal Data. All analyses were performed with Stata v14.1 (Stata-Corp, 2015, College Station, TX, USA).

The objective of the study was to evaluate the incidence of IgG antibodies to SARS-CoV-2 in patients diagnosed with lymphoma in medical oncology services and to describe the clinical characteristics of the positive patients.

## 3. Results 

The multicentre study, directed by the GOTEL group, included a total of 150 patients between 22 May and 11 June 2020. A total of seven hospitals assigned to the GOTEL group participated. The mean age at the time of taking the serology sample was 65 years (range 17–89 years), 70 patients were women (46.5%) and 80 men (53.5%). At the time of diagnosis of lymphoma, 13 cases were stage I (9%), 27 (18%) stage II, 37 (24.5%) stage III, and 73 (48.5%) stage IV, while 6.6% were of primary extranodal origin. The histological subtypes are described in the Table 1 and the active treatments (57 cases, 38%) at the time of the study are described in Table 2. A total of 10 cases with positive serology for SARS-CoV-2 were identified, which is a prevalence of 6% in this population. The distribution of cases studied by autonomous communities was as follows: Madrid, 43 cases (28.5%), of which 5 were IgG +; Catalonia 35 (23.5%) with 3 IgG + cases; Valencia, 64 (42.5%), with 2 positive cases; and the Canary Islands, 8 (5.5%), all of them with negative serology. The mean age in the positive patients group (10 cases) was 59 years (range 40–82 years), 60% men and all never smokers or ex-smokers (>1 year). Four cases corresponded to diffuse large B cell lymphoma (DLBCL), four follicular lymphomas (FL), one mucosa-associated lymphoid tissue (MALT), and one cutaneous T lymphoma. Two of them were on active treatment, one of them with chemotherapy (CHOP-R scheme) and one more with idelalisib. Only one patient required hospitalization and was managed with supportive treatment and intravenous corticosteroids; he did not require any invasive respiratory therapy or additional drugs. None of the patients required intensive care unit (ICU) management and all fully recovered from the infection; the clinical characteristics of these patients are described in Table 3.

## 4. Discussion

Lymphomas represent a wide spectrum of malignant neoplasms that can range from indolent forms to very aggressive forms, requiring intensive treatment in a short time [16]. The approach to these patients requires adequate study and management, including combined treatments that in many cases result in the cure of the disease but that also favour immunosuppression and may worsen the prognosis of SARS-CoV-2 infection. The COVID-19 pandemic has represented a major health problem for the general population and especially for some specific groups, such as elderly patients and patients with comorbidities, among which we highlight cancer [11,12,14,15]. Several studies have shown a negative impact of the pandemic in relation to the care of cancer patients. One of them assessed the impact of this pandemic on oncological care in the Netherlands, with more than 5300 patients. The most frequent changes included the adjustments of treatments (chemotherapy and immunotherapy) in 30% of the cases and the delay and discontinuation of treatment in 55% and 63% of the patients, respectively [17].

Since the beginning of the pandemic, attempts have been made to define risk groups and evaluate the different forms of presentation of the disease. The most frequently described symptoms are associated with respiratory infection, such as cough, fever, and dyspnoea. Other types of symptoms have been described to affect different organs, such as skin, sense organs (anosmia and dysgeusia), and even neurological ones [18]. In various series published, in patients with cancer and lymphomas, these symptoms are similar to those in the general population, although the severity may vary depending on the status and the type of the oncological disease and the treatments received [12,15].

Fatal events, associated with COVID-19 and risk factors, were analysed in a series of more than 1000 cancer patients in the UK, identifying populations more susceptible to developing severe disease. In this study, increasing age represents an independent factor for poor prognosis, as do haematological neoplasms (leukaemias, lymphomas, and myelomas) versus solid tumours [11]. Especially, those patients with high-grade leukaemia, whose natural evolution is rapid [19], and patients with bone marrow transplantation are the most vulnerable.

An Italian study with more than 500 patients infected with SARS-CoV-2 and with haematological neoplasms showed that 18% of the patients required ICU management. Fifty percent of the patients had a mild disease, 36% severe, and 14% critical. In univariate analysis, it was demonstrated that patients with severe or critical illness were older (*p* = 0.32), with a mean age of 68 years, with a higher Charlson comorbidity index (mean 5 vs. 4; *p* = 0.011) and a more recent diagnosis of haematologic disease [15]. The mortality rate in this series reached 37%. A retrospective and multicentre French study analysed a group of 89 patients infected with SARS-CoV-2 who had a recent or past diagnosis of lymphoma. The median age was 67 years (range, 19–92), 66% were men, and 72% had comorbidity. Eighty-six percent of the cases corresponded to non-Hodgkin B cell lymphomas. The patients were followed up for a median of 33 days, with a survival rate of 71% (95% CI, 62-81%). This study identified a higher risk of mortality in patients >70 years old (Hazard Ratio –HR- 2.87, *p* = 0.02) and a relapsed/refractory lymphoma (HR 2.54, *p* = 0.02). The 30-day overall survival was 88% (78–99%) for patients <70 years of age, without relapses/refractory lymphoma [20].

Although the data can be alarming, it is also important to carry out an adequate diagnosis and treatment of new haematological patients, since many of the treatments will have curative intent. For this, several scientific societies have developed guidelines for the management of patients with haematological malignancies to optimize treatment and minimize the risk of infection by SARS-CoV-2 [14,21,22,23]. Some of the most notable things are the efforts to avoid diagnostic and treatment delays, educate the patient on possible signs and symptoms of COVID-19, and perform diagnostic tests for the disease, according to local protocols.

A retrospective single-centre study of 18 patients, diagnosed with high-grade lymphomas during the pandemic, has shown that the use of standard treatment regimens is optimal and probably should not be modified due to a potential increased risk. This action makes it possible to give priority to the intensive management of patients to achieve their cure. Only one patient was infected with SARS-CoV-2 coinciding with an episode of febrile neutropenia [23].

Despite these efforts, no population studies have been conducted to determine the seroprevalence of SARS-CoV-2 and that is why our study is of significant relevance. In the 150 cases of patients with lymphomas analysed with a mean age at the time of taking the serology sample of 65 years old (range 17–89 years), we were able to determine a prevalence of IgG antibodies against SARS-CoV-2 of 6% in this population. In contrast to the national study of the seroprevalence of COVID-19 in Spain (ENE-COVID), which is 5% nationwide, we can say that despite the fact that the sample of patients with lymphomas is limited, the prevalence is similar to the general population [24]. The data from the ENE-COVID study were able to reliably determine the prevalence of COVID-19 both in the general population and in autonomous communities; however, the limitation of the number of cases per regions in our study does not allow us to make this estimation. The clinical characteristics of the patients in our series show that 80% of the cases had an asymptomatic or oligo-symptomatic course of the disease, with only one case of moderate severity that did not require treatment in the ICU.

In conclusion, the LYMPHO-COVID study is the first study of the seroprevalence of IgG antibodies in patients with lymphomas carried out in Spain, demonstrating a prevalence similar to the one of the general population. The data of infected patients does not appear to show greater aggressiveness of the infection or a worse clinical evolution than the general population, although there are few cases. To confirm our data, it is essential to carry out a study with a larger sample of patients.

## Figures and Tables

**Table 1:** Total cases according to the histological type and distribution of positive cases of IgG.

Type of Neoplastic	Nº	IgG +
Erdheim–Chester disease	1	0
Angioimmunoblastic T cell lymphoma	1	0
Splenic marginal zone lymphoma	1	0
MALT lymphoma	2	1
Primary cutaneous T cell lymphoma	6	1
Chronic lymphocytic leukaemia	6	0
Mantle cell lymphoma	7	0
Hodgkin lymphoma	27	0
Follicular lymphoma	42	4
Diffuse large B cell lymphoma	57	4
Total	150	10

MALT: extranodal marginal zone lymphoma.

**Table 2:** Distribution of treatments received by the patients.

Scheme	Nº (%)	Scheme	Nº (%)
CHOP-R	12 (31.5%)	ICE-Bendamustine	1 (2.6%)
Rituximab	10 (26%)	Liposomal doxorubicin	1 (2.6%)
Ibrutinib	8 (21%)	GEMOX	1 (2.6%)
ABVD	5 (13%)	CHOEP	1 (2.6%)
R-Bendamustine	5 (13%)	Obinutuzumab	1 (2.6%)
Nivolumab	2 (5.2%)	Venetoclax	1 (2.6%)
Obinutuzumab/Zanabrutinib	2 (5.2%)	CVP-R	1 (2.6%)
GEMOX-R	2 (5.2%)	Vemurafenib	1 (2.6%)
Ibrutumomab	1 (2.6%)	Radiotherapy	1 (2.6%)
ESHAP-R	1 (2.6%)	Total	57 (100%)

CHOP: Cyclophosphamide-Hydroxyldaunorubicin-Vincristine-Prednisone; R: Rituxumab; GEMOX: Gemcitabine-Oxaliplatin; ESHAP: Etoposide-Methylpredniolone-Cytarabine-Cisplatin; ICE: Ifosfamide-Etoposide-Carboplatin; CHOEP: Cyclophosphamide-Doxorubicin-Vincristine-Etoposide-Prednisone; CVP: Cyclophosphamide-Vincristine-Prednisone.

**Table 3:** Clinical characteristics of patients with positive serology (IgG) of SARS-CoV-2.

Case	Age	Gender	Histology	Stage	Extranodal Origen	ECOG	Comorbidities	Active Treatment	Scheme	Covid Diagnosis	Previous Antineoplasic Treatment	Treatment of Covid	Lymphoma Response
Patient 1	59	Female	DLBCL	IIE	Gastric	0	No	No	NA	Serology	CHOP-R	No	CR
Patient 2	60	Male	DLBCL	IIIS	No	0	No	No	NA	PCR	CHOP-R	Hospitalized *	CR
Patient 3	73	Female	FL	IV	No	0	COPD	No	NA	Serology	CVP-R	No	CR
Patient 4	41	Male	FL	IV	No	0	No	No	NA	PCR	R-monotherapy	Ambulatory	CR
Patient 5	65	Female	FL	IV	No	0	COPD	No	NA	Serology	R-monotherapy	No	CR
Patient 6	50	Male	C-TL	IE	No	0	No	No	NA	Serology	RT	No	CR
Patient 7	82	Female	MALT	IIE	Gastric	0	No	No	NA	Serology	Antibiotics	No	CR
Patient 8	64	Male	FL	IV	No	1	No	Yes	Idelalisib	Serology	CHOP-R	No	PR
Patient 9	58	Male	DLBCL	II	No	0	Diabetes	Yes	CHOP-R	Serology	NA	No	CR
Patient 10	69	Male	DLBCL	I	No	0	No	No	NA	Serology	CHOP-R	No	CR

DLBCL: Diffuse large B cell lymphoma; FL: Follicular lymphoma; C-TL: Cutaneous T lymphoma; COPD: Chronic obstructive pulmonary disease; CHOP: Cyclophosphamide-Hydroxyldaunorubicin-Vincristine-Prednisone; PCR: protein chain reaction; CR: Complete response; PR: Partial response. *The hospitalized patient did not require intensive therapy.

## Data Availability

In accordance with the journal’s guidelines, we will provide our data for the reproducibility of this study in other centers if such is requested. The Spanish Lymphoma Oncology Group is committed to responsible data sharing.
